# Supplementary material for: EWOD Chip with Micro-Barrier Electrode for Simultaneous Enhanced Mixing during Transportation
Source: Sensors (Basel). 2023 Aug 11;23(16):7102. doi: 10.3390/s23167102 (PMC10459807; doi:10.3390/s23167102)
Supplement: Supplementary file 1 [file sensors-23-07102-s001.zip › Supplementary Information.pdf]

Supplementary Information for

‘EWOD Chip with Micro-Barrier Electrode for Simultaneous Enhanced Mixing during Transportation’

Electrode design

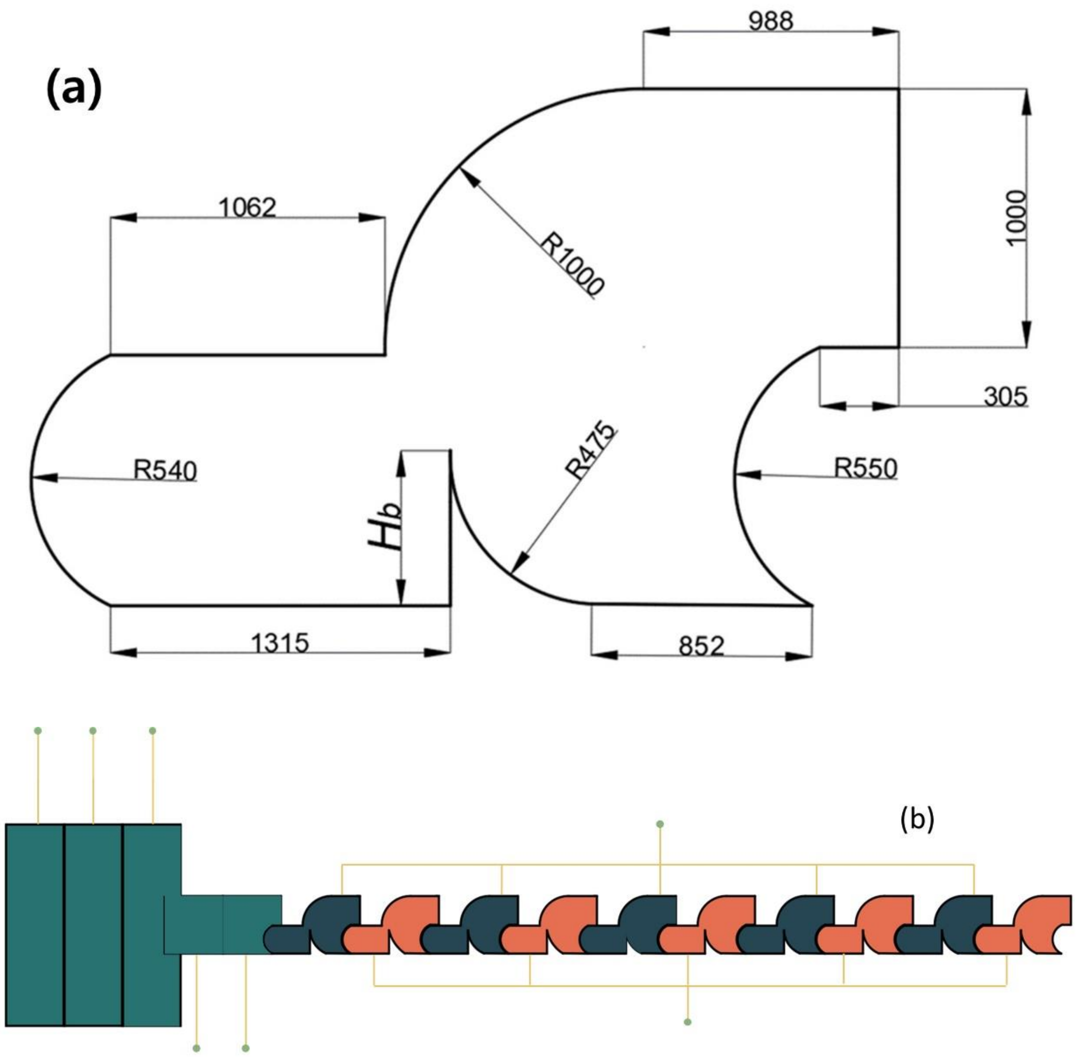

Figure S1. Schematic of electrode design and arrays.

Figure S1a shows our electrode design and the design of the array. In (b) the cyan rectangular electrode is used to generate microdroplets. The transport electrodes with microcarriers are embedded in it. The rectangular electrodes each have one electrical signal, while the transport electrodes with microcarriers require only two electrical signals for control.

Simulation results

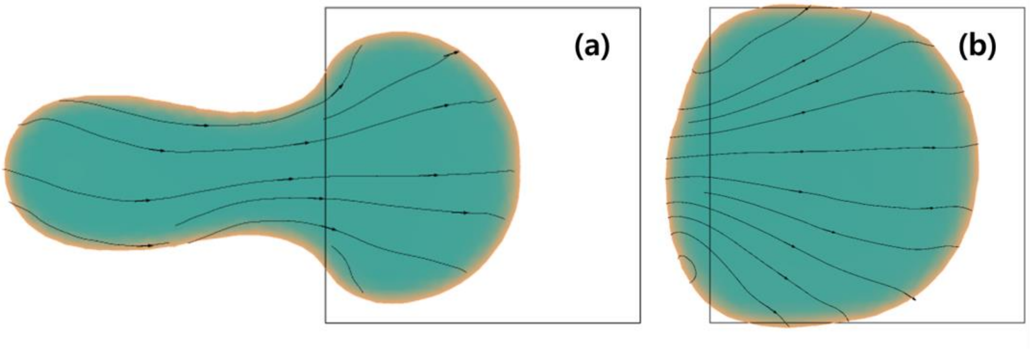

Figure S2. Internal streamline of the droplets on Re EWOD Chip.

Before conducting the experiments, simulations were first used to analyze the changes in the flow velocity within droplets on different electrodes. S2 shows the changes in flow velocity within droplets on a square electrode. It can be seen that during the motion, the flow velocity is mostly in the horizontal direction, which is the main reason for poor mixing effect on square electrodes.

Manufacturing of equipment

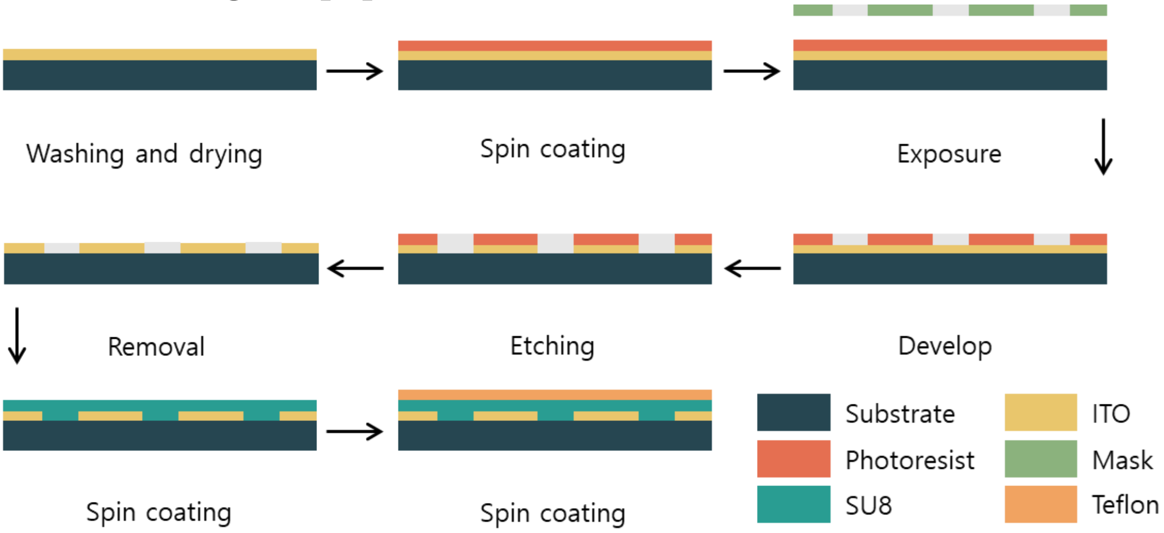

Figure S3. Preparation of lower plate.

A typical EWOD device consists of two poles, upper and lower, with a specific stopper inserted between them to ensure proper spacing. The upper and lower polar plates of the EWOD equipment are prepared using UV lithography and wet etching techniques in an ultra-clean room, which offers higher accuracy. Fig.S3 illustrates the preparation process of the lower plate, which requires only a layer of Teflon coating. The ITO material was purchased from 1, SU8 from 2, and Teflon from 3.

After the upper and lower polar plates were prepared, it was essential to verify that the contact angles of their surfaces met the experimental requirements. The contact angle $\boldsymbol{\theta}$ of the Teflon surface was measured at 121° using a contact angle measuring instrument. The relationship between the contact angle $\boldsymbol{\theta}$ and the applied voltage is illustrated in Figure S4C.

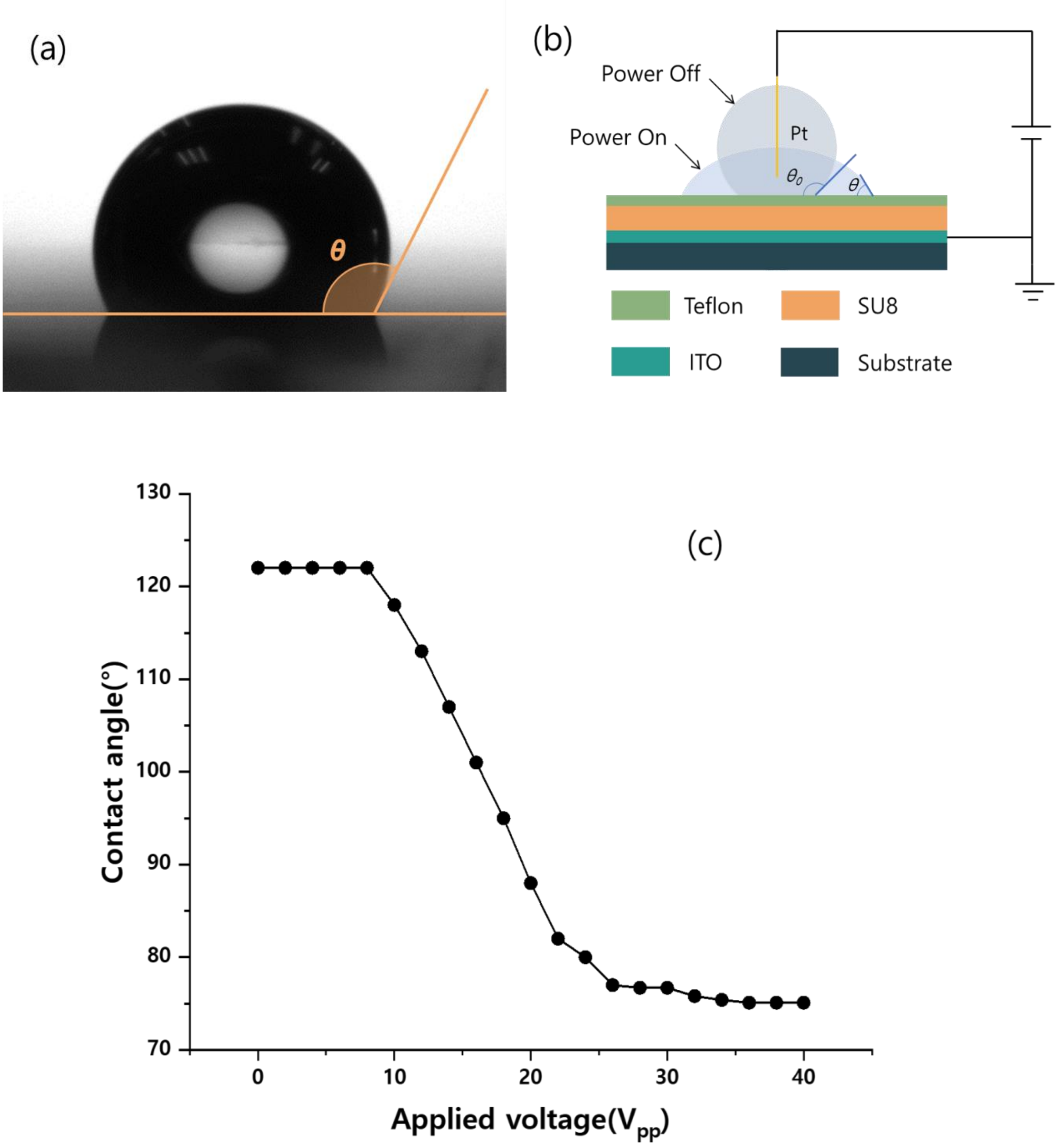

Figure S4. (a) The contact angle is expressed by $\boldsymbol{\theta}$. (b) Experimental setup. (c) $\boldsymbol{\theta}$ vs. Applied Voltage plots were generated for each experiment, with each experiment being conducted five times and an average value being calculated at the end. The experimental object used was 3 μL of deionized water, and the applied electrical signal had a frequency of 1 kHz in the form of an AC waveform.

Mixing and diffusion

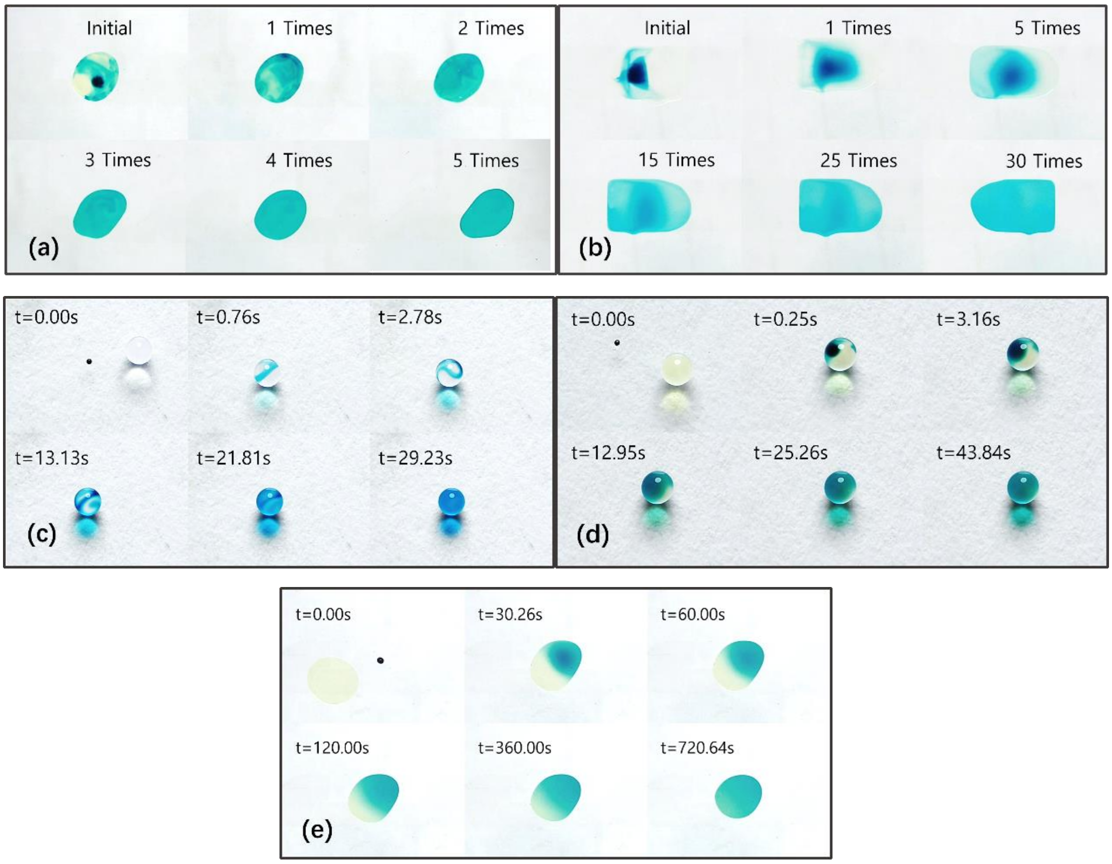

Figure S5. Spontaneous diffusion of internal dye in droplets in air. (a)Blue dye in yellow DI water mixing by Barrier EWOD Chip. (b) Blue dye in yellow DI water mixing by Re EWOD Chip. (c) Blue dye diffusion in DI water without in the plate. (d) Blue dye diffusion in yellow DI water without in the plate. (e) Blue dye diffusion in yellow DI water in the plate.

As a control group experiment, the mixing and diffusion results are shown in Figure S5. The rate of spontaneous diffusion is so low that it is understandable to ignore its effect on the experimental results.

Activation capability of transport electrodes with micro-barrier

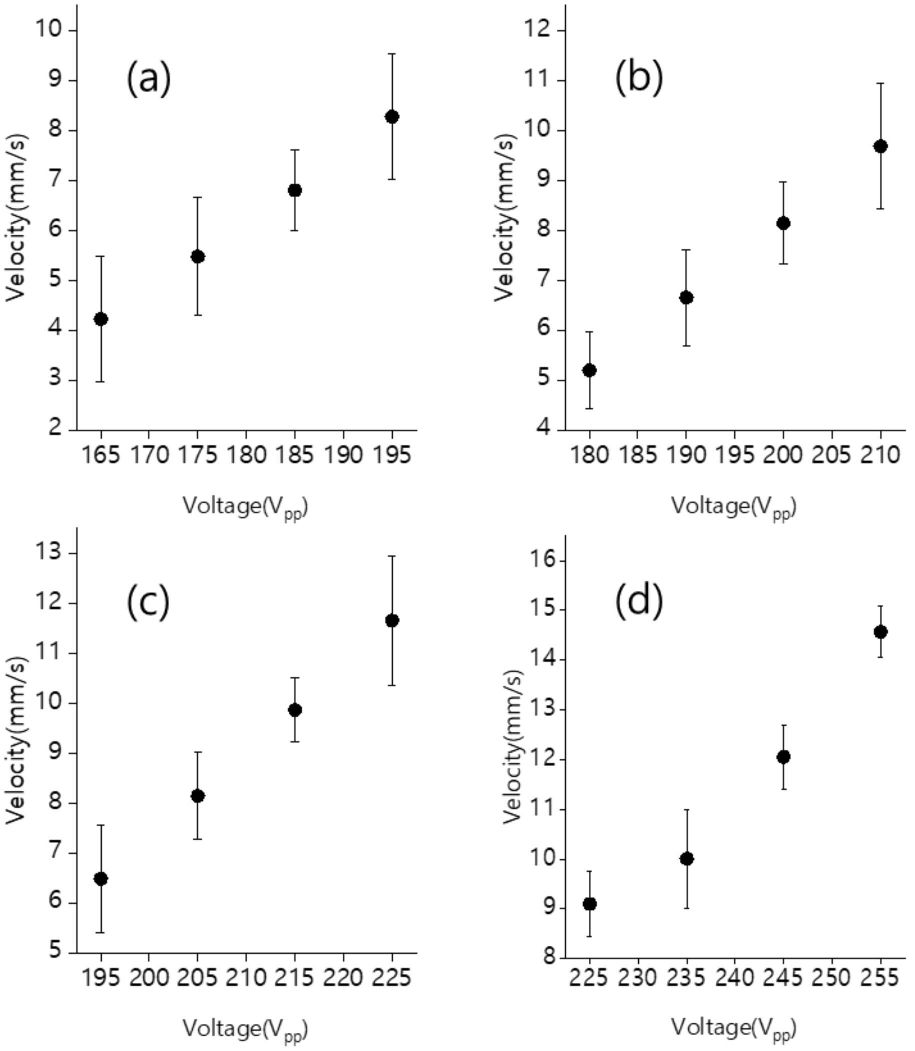

Figure S6. The activation rate for the electrodes was measured for different Hb cases, where (a)-(d) corresponded to $\boldsymbol{H}_b$ levels of 200, 400, 600, and 800 μm, respectively. It should be noted that the activation voltage threshold varied for different electrodes; therefore, in each group, the experiments were conducted above the threshold voltage's d-bottom line. The voltage frequency during all experiments was 1 kHz, the volume of deionized water is 0.65 μL.

Figure S6 illustrates the correlation between the applied voltage and the maximum droplet velocity for different $\boldsymbol{H}_b$ levels. The time taken for a successful droplet transfer from one electrode to the next was recorded experimentally and used to calculate the velocity. This velocity not only represents the transfer rate of the droplets, but also reflects the upper limit of the droplet mixing function's velocity. The experimental data mentioned above were fitted to determine that the velocity is proportional to the square of the voltage, which is consistent with our prior research as well as the $\boldsymbol{F}_{EWOD}$ formula.

Preparation of Mixing and Detection Solution for Sorbitol

The preparation process for the mixing and detection solution of sorbitol is as follows:

Step 1: Preparation of a sorbitol solution with a concentration of 2 mg/mL. The method of preparation involves adding 20 mg of sorbitol powder to 10 mL of DI water, followed by ultrasonic mixing for 10 min and incubation for 1 hour.

Step 2: Preparation of the detection solution. Firstly, place 0.5 μL of sorbitol solution on the platform, then add 0.15 μL of detection reagent one and two into the sorbitol solution.

Step 3: Mixing. The solution was mixed 0, 5, 15, and 30 times using the Barrier-EWOD chip and 5 times using the Re-EWOD chip. The amount of sorbitol was 0.5 μL, and 0.15 μL of both assay reagents I and II. The assay kits used in the experiments were purchased from *Beijing Solarbio Science & Technology Co*.

Step 4: Detection. Different mixed droplets with different mixing times are tested using a spectrophotometer (*V650 JASCO*). The absorption peak appears at 655nm.

Supplementary Movies

Movie S1 Spontaneous diffusion without operation (in a semiconductor wafer).

Movie S2 Rapid mixing on micro-barrier.

Movie S3 Transport of Double droplets on different $\boldsymbol{H}_{\mathbf{b}}$.
